# Multivariate analysis of soil particle size distribution and Spatial correlation with soil moisture characteristics in different vegetation types of Mu Us Sandy Land

**DOI:** 10.1038/s41598-025-10910-5

**Published:** 2025-07-15

**Authors:** Min Han, Yuefeng Guo, Chengfu Zhang, Wei Qi, Lu Cheng, Yajie Feng, Yang Song, Wenyuan Yang

**Affiliations:** 1 College of Desert Control Science and Engineering, Inner Mongolia Agricultural University, Hohhot, China; 2 Inner Mongolia Autonomous Region Water Conservancy Development Center, Hohhot, China

**Keywords:** Mu Us Sandy Land, Different vegetation types, Soil particle size distribution, Multiple fractal dimension, Soil Spatial moisture characteristic, Forestry, Restoration ecology

## Abstract

In this paper, the fractal mechanism of soil improvement by vegetation was revealed by analyzing the soil characteristics under four typical vegetation types: *Salix cheilophila*, *Caragana korshinskii*, *Hippophae rhamnoides*, and *Corethrodendron fruticosum* in Mu Us Sandy Land. The results showed that (1) the soil of each vegetation type was mainly composed of sand (> 90%), and the content of clay and silt was less than 10%. CF had the least heterogeneity of soil particles in the 0–120 cm soil layer, HR was uniformly distributed in the 120–200 cm soil layer, and the change trend of particle composition in the 140–200 cm soil layer was consistent. (2) Soil fractal dimension (D) was positively correlated with soil clay and silt content, and negatively correlated with sand content. The generalized dimension spectrum D(q) decreases in the “S” type, and the sensitivity was higher in the region of q < 0, indicating that the particle distribution in the sparse area is more susceptible to disturbance. (3) HR had the best water holding capacity in the surface layer (0–40 cm) and deeper layer (120–200 cm), and the average soil water storage in 0–200 cm reached 791.61 mm (13.76% higher than CF). All vegetation had soil water deficit in specific soil layers, among which SC had water deficit in 20–30 cm, CK in 0–10 cm, HR in 60–80 cm, and CF in 180–200 cm soil layers (0.91 mm, 0.90 mm, 0.92 mm, and 0.93 mm). (4) HR significantly affected soil bulk density and porosity by increased silt and sand (*P* < 0.05). The fractal parameters of SC were significantly correlated with soil water content (*P* < 0.05). The pH of CK was a significant correlation with soil water storage (*P* < 0.01). CF soil particle composition and fractal parameters were significantly correlated with soil moisture content, bulk weight, and capillary porosity (*P* < 0.05). The study showed that HR achieves the best water retention and soil modification effect by optimizing soil structure and is the preferred vegetation for ecological restoration of the Mu Us Sandy Land.

## Introduction

Mu Us Sandy Land is located in the transition zone from Ordos Plateau to Loess Plateau and is situated in the intertwined zone of agriculture and animal husbandry in the north of China, and irrational exploitation and utilization by human beings have led to further deterioration of desertification in the Mu Us Sandy Land^1^. In order to improve the environment, people have implemented vegetation restoration measures such as fly-seeding afforestation (*Salix cheilophila*, *Caragana korshinskii*, *Hippophae rhamnoides*, and *Corethrodendron fruticosum*) in the Mu Us Sandy Land and have achieved good results^2^. The root system of these plants is extremely developed and can firmly fix the sandy soil and increase the structural stability of the soil. At the same time, they are able to intercept rainwater and reduce surface runoff, which helps to maintain soil moisture in sandy areas. In addition, the root system of these plants can loosen the soil during the growth process and improve the physical properties of the soil^3^. Different vegetation restoration measures have a significant effect on soil quality in sandy areas and are a key step in restoring the ecosystem of the Mu Us Sandy Land areas using vegetation measures^4^.

Soil is an irregular, complex, and porous medium composed of different particles, with a self-similar structure and significant fractal characteristics^5^. The fractal dimension can be divided into single and multiple fractal dimensions. The single fractal dimension can characterize the roughness of the overall soil^6^, while the multiple fractal dimensions can reflect the local characteristics of the soil particle distribution more accurately^7^. Du et al.^8^ used the single fractal dimension to analyze the fractal characteristics of soil particles in different vegetation patches and found that it was significantly positively correlated with pH and EC, but the single fractal dimension had limitations and could not reflect the local characteristics and variation of soil particle distribution^9,10^. In contrast, the multiple fractal dimension can not only describe the non-homogeneity of soil particle distribution more comprehensively but also quantitatively characterize the spatial heterogeneity of its physical properties. In addition, the water conservation capacity of the soil layer, as the core service function of terrestrial ecosystems, directly affects the regional climate, hydrological conditions, vegetation distribution, and soil condition, and is a key indicator for assessing ecosystem health^11^. Studies have shown that vegetation type significantly affects water conservation-related indicators such as soil moisture content, soil bulk density, soil water storage, and water deficit, and there are significant differences in soil physical properties under different vegetation covers^12,13^. Yuan et al.^14^ further confirmed that soil water holding capacity was negatively correlated with bulk density and sand content in the study of vegetation in the Qilian Mountains. This finding has important guiding significance in the study of soil moisture dynamics in ecologically fragile areas, such as the Mu Us Sandy Land.

The existing studies in the Mu Us Sandy Land are mostly focused on the spatial pattern of shrub communities^15^, the temporal and spatial dynamics changes of land^16^, and the analysis of plant leaf traits^17^. However, there is still a gap in the research on the fractal characteristics of soil particles and their association with spatial patterns of moisture. In order to reveal the interactions between fractal characteristics of soil particles and water dynamics under different vegetation types, four typical vegetation types, *Salix cheilophila*, *Caragana korshinskii*, *Hippophae rhamnoides*, and *Corethrodendron fruticosum*, were selected in this study. Through fractal theory and multi-dimensional water analysis, the purpose of this study was to (1) Clarify the composition of the soil particles of the four vegetation types and the characteristics of single and multiple fractal dimensions; (2) Analyze the pattern of change of soil moisture and its correlation with fractal dimensions; and (3) Evaluate the effect of vegetation types on the relationship between soil particle size and water content. The results of the study can provide quantitative theoretical support for vegetation configuration and soil improvement in the region.

## Materials and methods

### Overview of the study area and soil sample collection

The study area is located in the Mu Us Sandy Land, Yijinhuoluo Banner State-owned Forest Farm Xinjie Sand Control Station under the Taigemiao 68 operating area Maogaitu woodland (39°10′30″ N, 109°35′47″ E), at an altitude of 1388 m. The area belongs to the typical temperate continental semi-arid climate; the annual average temperature is 6.0–8.5 °C, the annual precipitation is 250–440 mm, and the soil is dominated by aeolian sandy soil^18^. The main vegetation is dominated by The main vegetation types include *Salix cheilophila*, *Caragana korshinskii*, *Corethrodendron fruticosumvar*, *Artemisia ordosica*, *Corethrodendron scoparium*, and other shrubs that are dominant^19^.

In August 2024, *Salix cheilophila*, *Caragana korshinskii*, *Corethrodendron fruticosumvar*, *Artemisia ordosica*, and *Corethrodendron scoparium* were selected as the study objects, and four sample plots were set up. The characteristics of different vegetation types are shown in Table 1. Three plants of the same size were selected from each of the four plots. The sampling depth of soil samples was determined according to the depth of vegetation roots in the area, and the sampling depth was 200 cm. The soil layers were divided into 0–10 cm, 10–20 cm, 20–30 cm, 30–40 cm, 40–60 cm, 60–80 cm, 80–100 cm, 100–120 cm, 120–140 cm, 140–160 cm, 160–180 cm, and 180–200 cm for each sampling. The undisturbed soil was collected with a ring knife with a volume of 100cm^3^, and the disturbed soil was sealed with a plastic bag. The soil particle size was determined by the Malvern Mastersizer 2000 M laser particle size analyzer. 0.5 g of soil samples were selected and placed in a beaker, and hydrogen peroxide and hydrochloric acid were added sequentially to remove organic matter and carbonate. According to the method of the United States Department of Agriculture (USDA), the soil particle composition was categorized into clay (0–2 μm), silt (2–50 μm) and sand (50–2000 μm)^20^. Soil bulk density and saturated water content were measured by the ring knife method^21^.

**Table 1:** Sampling sites of different vegetation types.

Plant type	Location		Altitude/m	Average height/m	Crown width/m	Root depth/m
	Latitude	Longitude				
SC	39°18′66″	109°59′80″	1375	2.33	4.63	1.85
CK	39°19′02″	109°59′26″	1390	1.41	1.72	2.20
HR	39°18′56″	109°59′66″	1374	2.73	2.97	1.22
CF	39°18′95″	109°59′20″	1389	1.64	3.68	2.67

SC: Salix cheilophila; CK: Caragana korshinskii; HR: Hippophae rhamnoides; CF: Corethrodendron fruticosum.

### Calculation of soil Spatial moisture

1. Soil water content^22^:
  $$\:\text{S}\text{W}\text{C}=\frac{\left({\text{m}}_{2}-{\text{m}}_{1}\right)}{\left({\text{m}}_{1}-{\text{m}}_{0}\right)}$$

Where SWC refers to soil water content (%), m_0_ refers to the weight of the loop cutter (g), m_1_ refers to the weight of the loop cutter and dried soil sample (g), and m_2_ refers to the weight of the loop cutter and fresh soil sample (g).

(2) Soil bulk density:
  $$\:\text{B}\text{D}=\frac{\left({\text{m}}_{1}-{\text{m}}_{0}\right)}{\text{V}}$$

Where BD refers to the soil bulk density (g/cm^3^), m_0_ refers to the weight of the loop cutter (g), m_1_ refers to the weight of the loop cutter and the dried soil sample (g), and V refers to the volume of the loop cutter (100cm^3^).

(3) Soil capillary porosity:
  $$\:\text{P}\text{c}=\frac{{\text{W}}_{8\text{h}}-{\text{W}}_{105 \circ\text{C}}}{\text{V}}\times\:100{\%}$$

Where W_8h_ is the weight of the loop cutter after 8 h of immersion in water (g), W_105°C_ is the constant weight of the loop cutter (g), and V refers to the volume of the loop cutter (100cm^3^).

(4) Soil water storage^23^:
  $$\:\text{S}\text{W}\text{S}=0.1\times\:{{\uptheta\:}}_{\text{m}}\times\:{\text{B}\text{D}}_{\text{i}}\times\:\text{d}$$

Where SWS is the soil water storage capacity (mm), the measured mass moisture content of the layer (%), the soil bulk density of the layer (g/cm^3^), and d is the thickness of the soil layer (cm).

(5) Soil water deficit:
  $$\:\text{D}\text{S}\text{W}\text{i}=\frac{{\text{F}}_{\text{i}}-{\text{W}}_{\text{i}}}{{\text{F}}_{\text{i}}}\times\:100{\%}$$

Where DSW_i_ is the soil water deficit in each measurement layer (%); F_i_ is the field water holding capacity (mm) in each measurement layer; and W_i_ is the soil water storage capacity (mm) in each measurement layer.

### Fractal dimension of soil particle size distribution

The fractal dimension (D) was calculated using the fractal model derived by Tyler. Based on soil particle size volume fraction data with the following Eq.^9^:

$$\:\frac{V(r<{R}_{i})}{VT}={\left(\frac{{R}_{i}}{RL}\right)}^{3-D}$$

Where: r is the soil particle size, $\:{R}_{i}$ is the particle size of level i, $\:\text{V}(\text{r}<{\text{R}}_{\text{i}})$ is the volume of particle sizes smaller than, VT is the total volume, and RL is the largest particle size. Taking logarithms on both sides of the above equation, the log$\:\frac{\text{V}(\text{r}<{\text{R}}_{\text{i}})}{\text{V}\text{T}}$ and log$\:{\left(\frac{{\text{R}}_{\text{i}}}{\text{R}\text{L}}\right)}^{3-\text{D}}$ were performed with linear fitting for vertical and horizontal coordinates; 3-D is the slope, and D is the soil fractal dimension.

### Multiple fractal dimensions of soil particle size distribution

In order to gain more insight into additional information about the distribution of soil particle sizes, we use the method proposed by Chhabra^24^, which determines f(α) (the singularity spectrum of the multivariate) directly from the experimental data. Compared to the D-value, the multivariate dimension provides a more complete picture of the morphological and structural properties within the soil as well as the complexity and uniform distribution of the soil structure. Based on soil particle size measurements, the analytical results were obtained in the interval I = [0.02, 2000], which is the volume percentage of soil particle sizes corresponding to 100 sub-intervals (particle size segments). We divided the interval I into equal-length intervals (I_i_[φ_i_, φ_i+1_] (i = 1,2,3,.)), where φ_i_ represents the diameter of soil particle sizes. In addition, each partition has a total of N_(ℇ)_ intervals of the same size, J = 2^K^ (K = 1,2,3,4,5,6). In addition, for each partition, the probability of soil particle size distribution is calculated as follows:

$$\:{{\upmu\:}}_{\text{i}}\left({\upepsilon\:}\right)=\frac{{\text{V}}_{\text{i}}}{{\text{V}}_{\text{T}}}$$

where: and refer to the volume occupied by soil particle sizes in part i and the total volume, respectively.

The generalized dimension D(q) of the multiple dimensions of the soil particle size distribution is given by^25^:

$$\:\text{D}\left(\text{q}\right)=\underset{{\upepsilon\:}\to\:0}{\text{lim}}\frac{1}{\text{q}-1}\frac{\text{lg}\left(\sum\nolimits_{\text{i}=1}^{{\text{N}}_{\left({\upepsilon\:}\right)}}{{\upmu\:}}_{\text{i}}{\left({\upepsilon\:}\right)}^{\text{q}}\right)}{\text{log}{\upepsilon\:}}\left(\text{q}\ne\:1\right)$$

$$\:\text{D}\left(1\right)=\underset{{\upepsilon\:}\to\:0}{\text{lim}}\frac{{{\upmu\:}}_{\text{i}}\left({\upepsilon\:}\right){\text{log}}_{{{\upmu\:}}_{\text{i}}}\left({\upepsilon\:}\right)}{\text{log}{\upepsilon\:}}\left(\text{q}=1\right)$$

Represents the structural complexity and heterogeneity of soil particle size distribution (-10 ≤ q ≤ 10). Here D_0_, D_1_, and D_2_ are the capacity dimension, information dimension, and correlation dimension for q = 0, 1, and 2, respectively; D_0_ is the capacity dimension, the larger the value, the wider the distribution range of soil particle sizes; D_1_ is the information dimension, the larger the value, the more concentrated the distribution of particle sizes; D_2_ is the correlation dimension, the larger the value, the more uniform the interval of the particle size measurements.

### Data processing and analysis

Data were organized and calculated using Excel 2016. One-way analysis of variance (ANOVA) with a probability less than 0.05 was performed on the selected indicators using SPSS 26 software to determine the differences between the same soil strata of different vegetation types as well as between different soil strata of the same vegetation. Matlab R2024a was used for plotting the generalized dimension spectrum and calculating the fractal and multivariate dimension parameters. Pearson correlation analysis was used to study the correlation between soil particle size composition, fractal parameters (clay, silt, sand, D_1_, D_2_, D_1_/D_2_, and D_1_*D_2_) and soil environmental factors (soil water content, bulk density, capillary porosity, water storage, and water deficit). Plots were made using Origin 2024b.

## Results and analysis

### Soil particle size distribution of different vegetation types

#### Soil particle size distribution and single fractal dimension

**Fig. 1:**
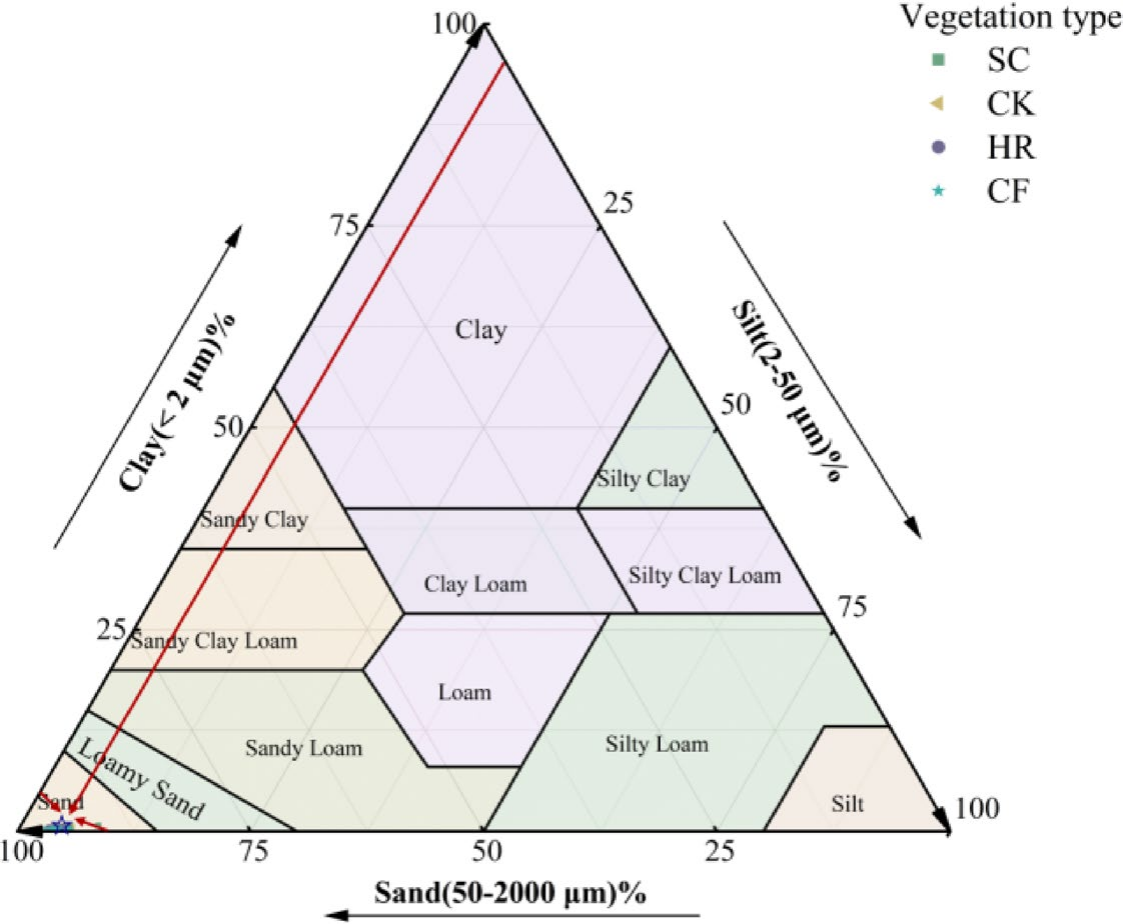
Soil texture classification of different vegetation types.

**Fig. 2:**
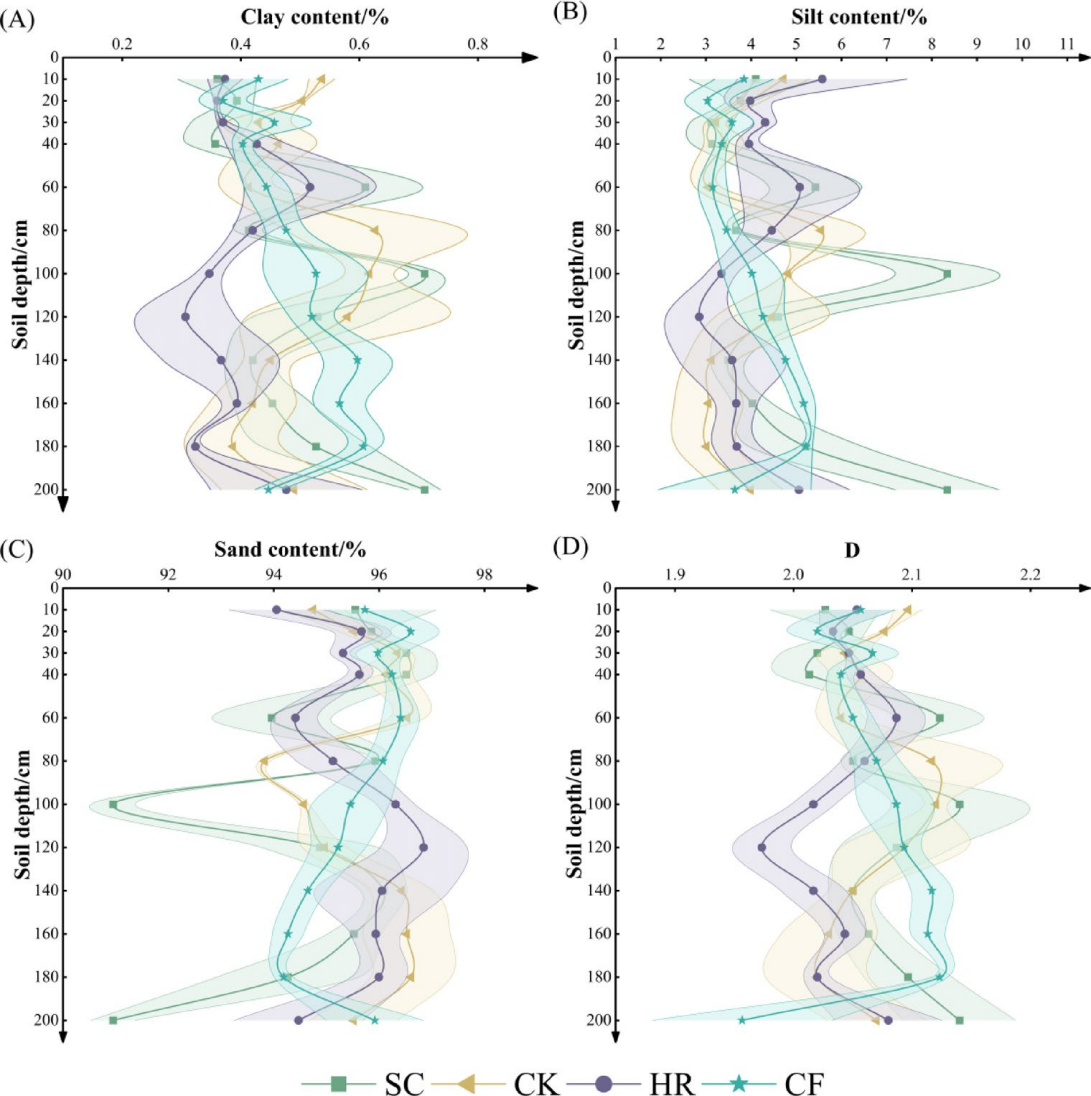
Soil particle size composition and fractal dimension of different vegetation types.

According to the International Standard for Soil Texture Classification (ISSC), the soil texture type in the study area is mainly characterized by sandy grains (Fig. 1). As can be seen in Fig. 2, SC clay content was higher at 180–200 cm, reaching 0.71%, while it was relatively lower at 0–40 cm soil depth, ranging from 0.36 to 0.39%. CK content was relatively higher at 0–10 cm (0.54%). HR content was relatively smooth, mostly ranging from 0.31 to 0.43%, although there was a relatively high value of 0.52% at 40–60 cm. There was a relatively high value of 0.52% at 40–60 cm. CF silt content ranged from 0.36 to 0.53% at 0–20 cm starting from the 20–40 cm soil layer and increasing with soil depth. Silt varied somewhat among the different vegetation types, with values ranging from about 2.0–10.0%. SC the highest value of silt content was reached at 80–100 cm, while the other vegetation types had lower content at this soil depth. Sand content was generally high across vegetation, all above 90%. The four vegetation types showed the same trend at 140–200 cm, decreasing with increasing soil depth. SC had slightly lower sand content than the other vegetation types at soil depths of 80–100 cm and 180–200 cm, but overall, the sand content was relatively similar across vegetation.

The range of D-values under different vegetation types was roughly between 1.96 and 2.14 with relatively small differences. SC had a relatively high D-value (2.14) at 80–100 cm, which was higher than that of other vegetation. CF had the smallest D content (1.96) at 180–200 cm. The trend of D-value varied with soil depth in different vegetation. HR showed a gradual increase in D-value in the shallow soil layer (0–40 cm); CF showed a gradual increase in the middle soil layer (40–140 cm); CF showed a slight decrease in the deeper soil layer (140–200 cm), while the other three vegetation showed an increasing trend in deeper soil layer. Under the four vegetation types, the soil particle size in clay, silt, and D-value in different soil layers had the same distribution trend, and the opposite trend in sand, indicating that the D-value was positively correlated with the content of soil clay and silt particle sizes, and negatively correlated with the content of sand.

#### Multiple fractal dimensions of soil particle size distribution of different vegetation types

**Fig. 3:**
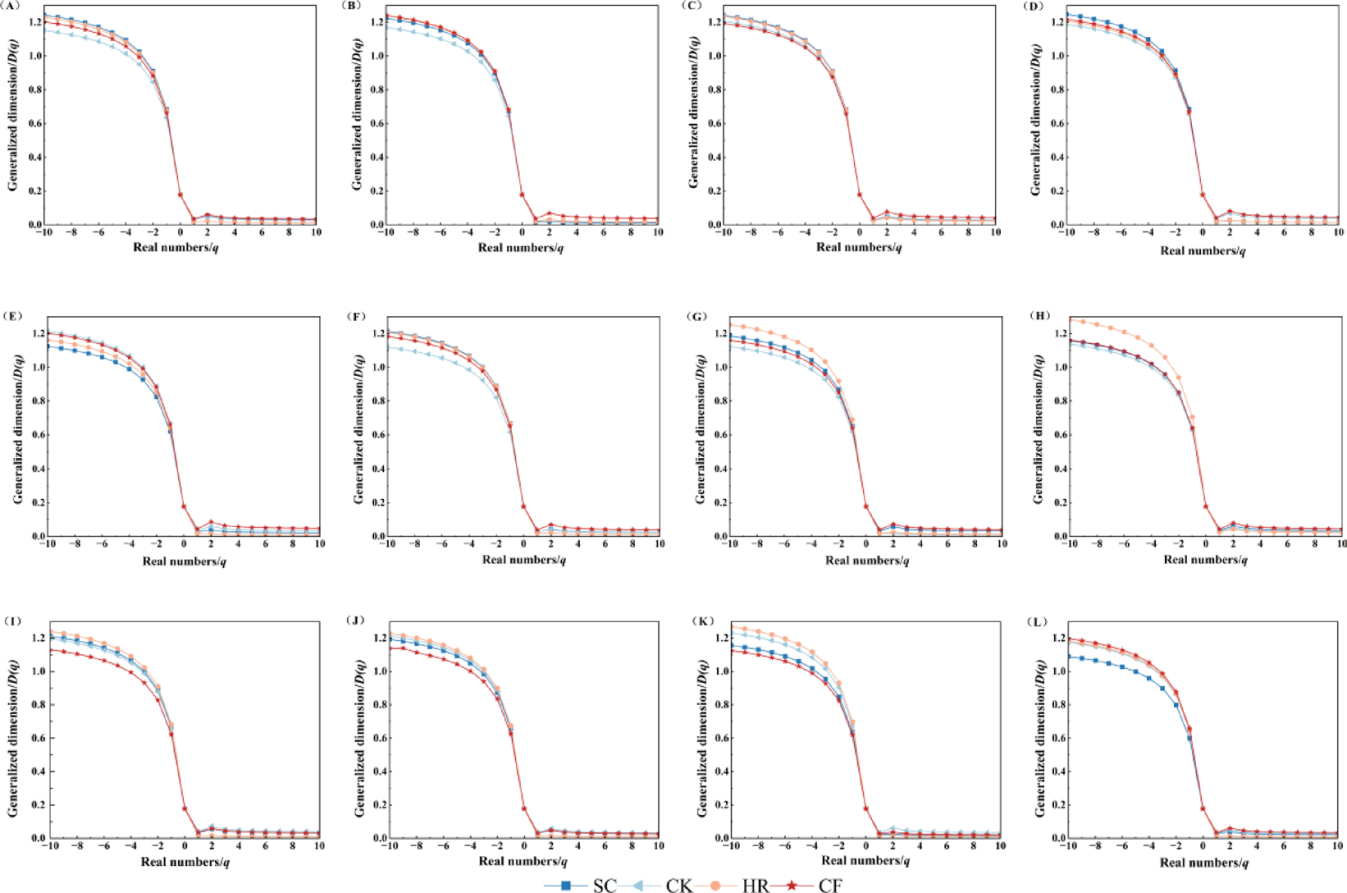
Generalized dimensional spectrum of soil particle size distribution D(q)-q curves for different vegetation types. (**A**), 0–10 cm; (**B**), 10–20 cm; (**C**), 20–30 cm; (**D**), 30–40 cm; (**E**), 40–60 cm; (**F**), 60–80 cm; (**G**), 80–100 cm; (**H**), 100–120 cm; (**I**), 120–140 cm; (**J**), 140–160 cm; (**K**), 160–180 cm; (**L**), 180–200 cm.

The generalized dimensional spectra of soil particle size distribution under different vegetation types showed an inverse “S” shape decline (Fig. 3), which can be described by an S-shaped curve. For the soil particle size distribution under four vegetation types, the gradient decrease of the generalized dimensional spectrum D(q) was significantly larger at q < 0 than at q > 0, indicating that D(q) was more sensitive in the sparse region. When q > 0, we found smaller values of variation across vegetation, with soil particle size distribution CF having the highest D(q) values in the 0–120 cm and 180–200 cm soil horizons, and CK having the highest D(q) values in the 120–160 cm soil horizons, suggesting that soil heterogeneity is large in this soil horizon corresponding to vegetation. When q < 0, CK had the lowest D(q) values at 0–20 cm, 30–40 cm and 60–120 cm; CF had the lowest D(q) values at 20–30 cm and 120–180 cm; and SC had the lowest values at 40–60 cm and 180–200 cm, indicating large soil non-uniformity.

**Fig. 4:**
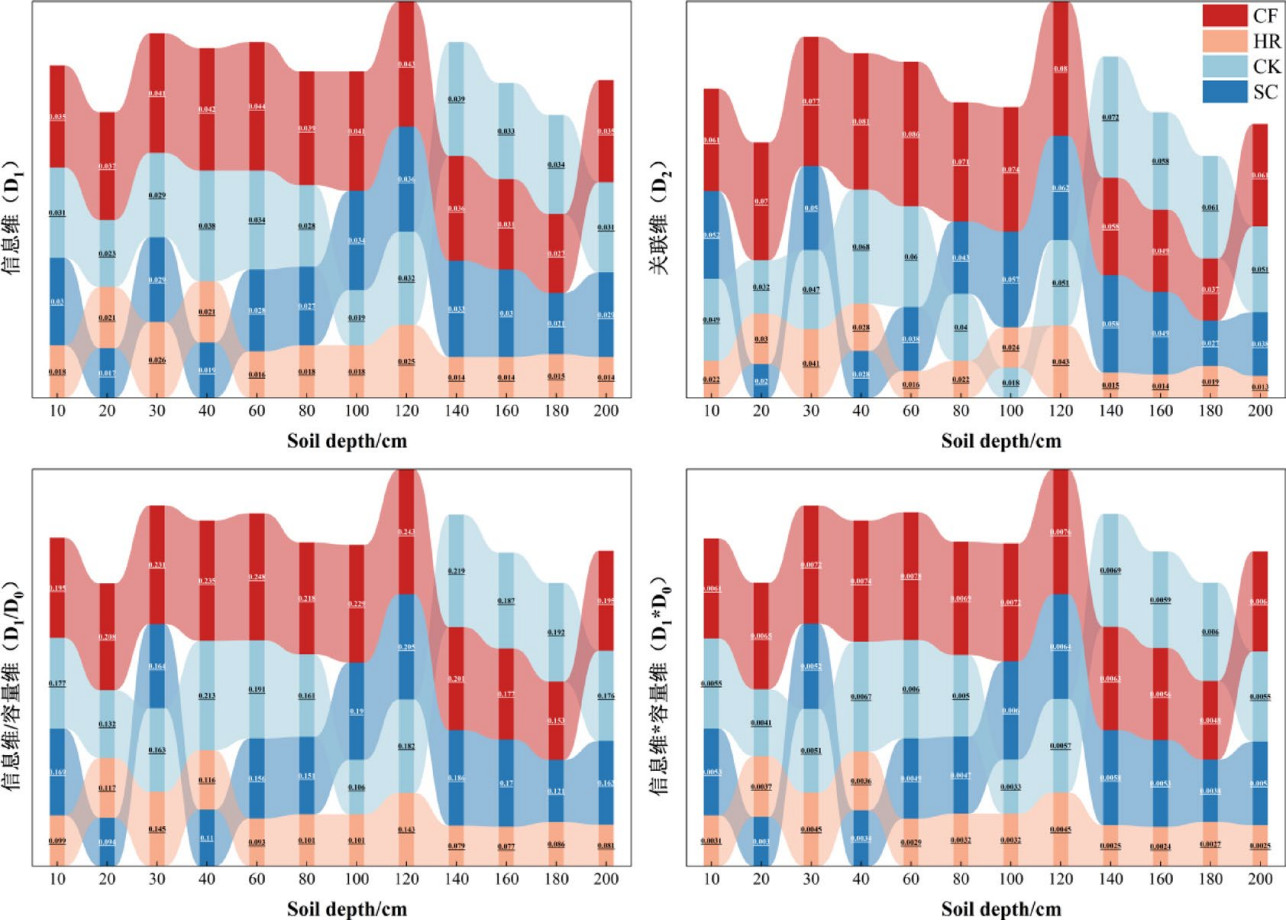
Multiple fractal dimensions characteristics of soil particle sizes in different vegetation types.

D_0_, D_1_, D_2_, D_1_/D_0_, and D_1_*D_0_ were extracted from the generalized dimensional spectrum (Fig. 4). D_0_ indicates the density of continuous distribution of soil particle sizes, the larger the value, the wider the distribution of soil particle sizes, and the D_0_ of all four vegetation types was 0.177, indicating that they had the same range of distribution. D_1_ and D_2_ indicate the degree of concentration of the distribution of soil particle sizes and the variability of the soil particle sizes, the greater the value representing the distribution of the particle sizes was uneven and the smaller the variability. In the 0–120 cm soil layer, D_1_ and D_2_ of CF were larger than those of SC, CK, and HR, indicating that the distribution of CF soil particle sizes in this soil layer was uneven and the variability of particle size was low. In the 120–200 cm soil layer, the differences between SC, CK, and CF were not obvious, and HR was lower than other vegetation types, indicating that the distribution of HR soil particle sizes in this soil layer was concentrated and particle size variability was high. D_1_/D_0_ can be used to measure the degree of dispersion of the distribution of soil particle sizes, and the closer the value is to 1, it indicates that the distribution of soil particle sizes is more concentrated in the dense area, and the closer the value of D_1_/D_0_ is to 0, D_1_/D_0_ values for the four vegetation types ranged from 0.077 to 0.248, which indicates that soil particle size distribution is mostly concentrated in sparse areas. The smaller the value of D_1_*D_0_, the higher the uniformity of soil particle sizes concentrated in the area. Since the value of D_0_ of four vegetation types is the same in different soil layers, it has the same trend as that indicated by D_1_, presenting a lower uniformity of CF than that of the other vegetation types.

### Characteristics of soil spatial moisture characteristic in different vegetation types

#### Changes in soil Spatial moisture characteristics of different vegetation types

**Fig. 5:**
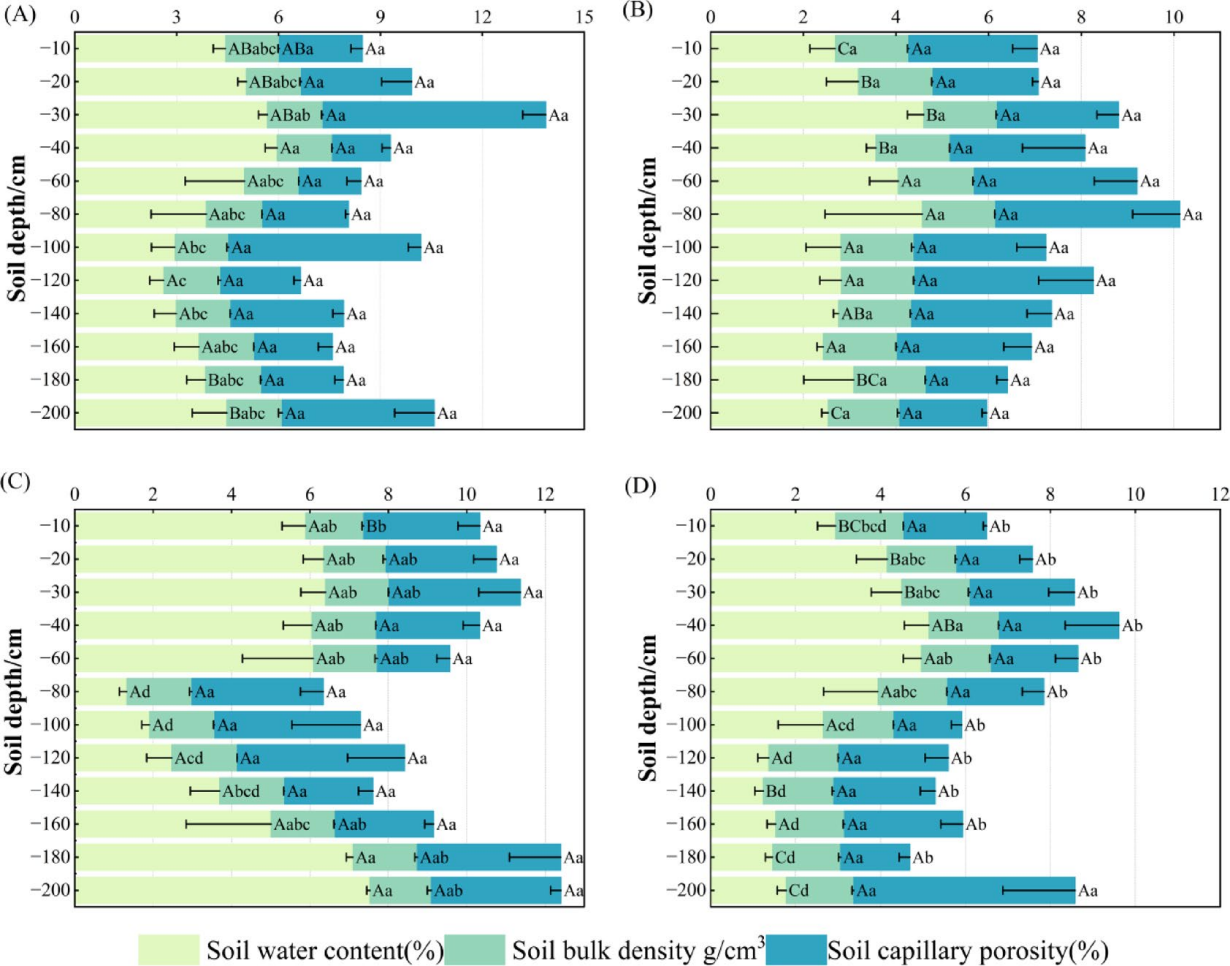
Changes in soil water content, soil bulk density, and soil capillary porosity in different vegetation types. Different uppercase letters indicate significant differences (*P* < 0.05) between the same soil depths for the four vegetation types, and different lowercase letters indicate significant differences (*P* < 0.05) between different soil layers for the same vegetation.

As can be seen in Fig. 5, the water content of the four vegetation types differed significantly at some soil layers (*P* < 0.05), showing HR > SC > CF > CK at 0–40 cm; HR was significantly higher than the other vegetation types at 160–200 cm; SC, HR > CK > CF at 120–140 cm, and the rest of the soil layers were not significant (*P* > 0.05). Tolerance was significant only at 0–10 cm, showing a trend of CK > CF > SC > HR. Soil capillary porosity was not significant at 0–200 cm (*P* > 0.05). Soil water content of SC was significant among different soil layers and was highest at 30–40 cm (*P* < 0.05), showing a trend of increasing, then decreasing, and then increasing, respectively; Soil water content and soil capillary porosity were not significant (*P* > 0.05). Soil water content, soil bulk density, and soil capillary porosity were not significant in CK (*P* > 0.05). HR the soil water content and soil bulk density varied significantly (*P* < 0.05), with the highest soil water content at 160–200 cm, consistent with the pattern of SC; The soil bulk density was the highest at 60–140 cm.The soil water content of CF was significant (*P* < 0.05), with a tendency of increasing and then decreasing; The soil bulk density and the soil capillary porosity were not significant (*P* > 0.05).

#### Soil water storage characteristics of different vegetation types

**Fig. 6:**
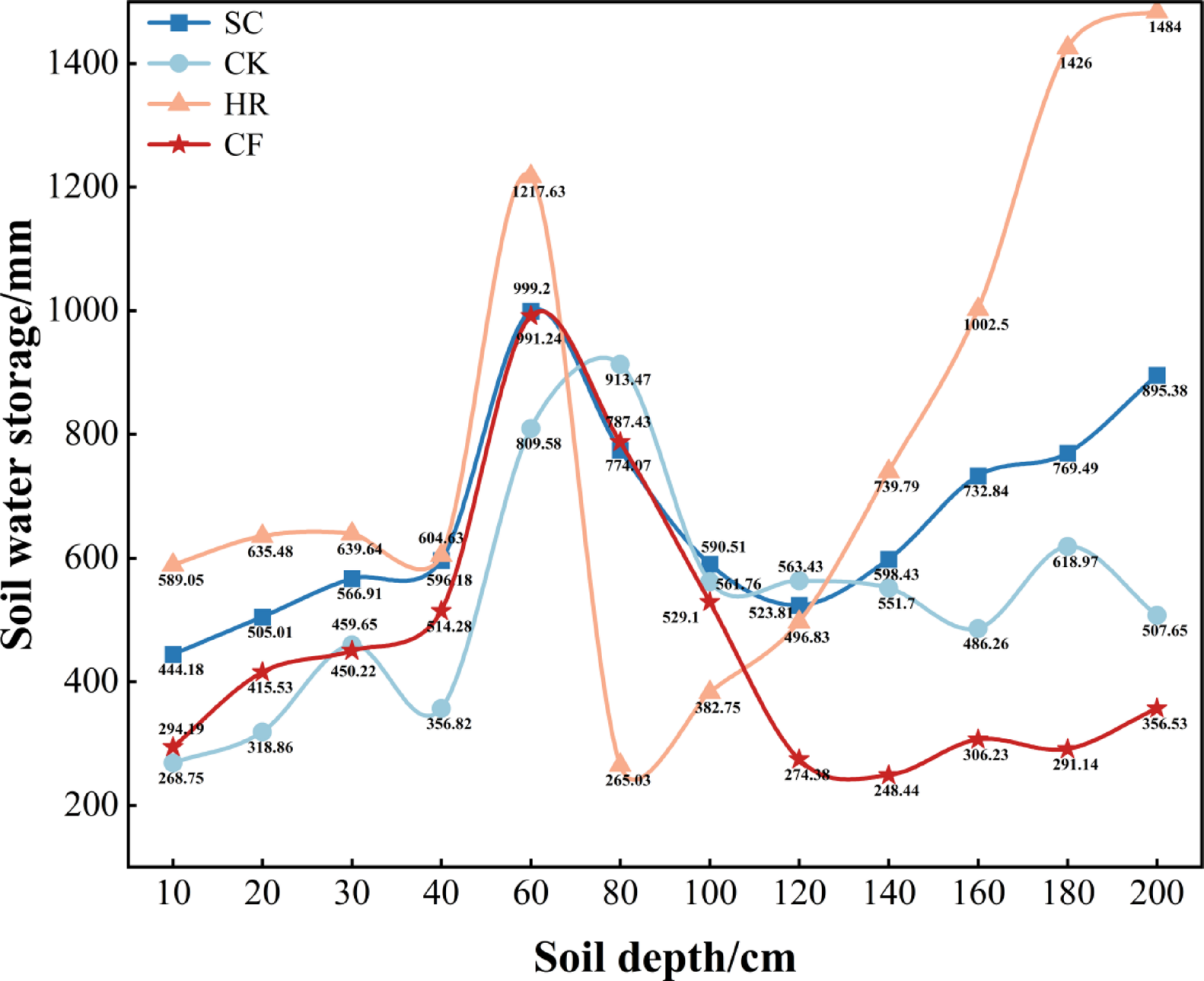
Changes in soil water storage in different vegetation types.

As shown in Fig. 6, the differences in soil water storage among different soil layers of the four vegetation types reflected the characteristics of soil water storage or depletion during the year, and the soil water storage of SC, CK, HR and CF ranged from 250 mm to 1500 mm in the depth range of 0–200 cm, and the average soil water storage of the four vegetation types was in the following order: HR (791.61 mm) > SC (666.34 mm) > CF (534.74 mm) > CK (454.89 mm). It can be seen that the HR zone had the highest soil water storage, which was 13.76% higher than the CF zone. It can also be seen that the four vegetation types consistently showed a trend of increasing, then decreasing, and then increasing with the increase of soil layers, and the HR peaked at 0–60 cm and 120–200 cm, CK at 60–80 cm, and 100–120 cm, and SC at 80–100 cm layers.

#### Soil water deficit characteristics of different vegetation types

**Fig. 7:**
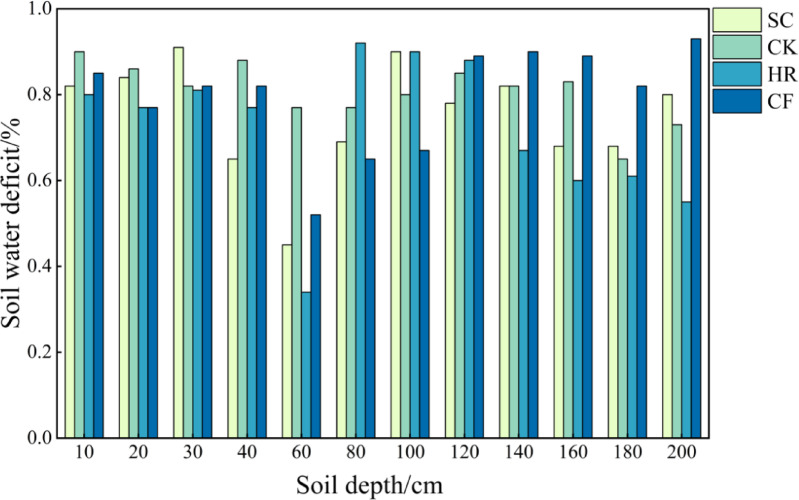
Soil water deficit in different vegetation types.

As shown in Fig. 7, the soil water deficit of the four vegetation types in the 0–200 cm soil layer was 0.74 mm, 0.81 mm, 0.72 mm and 0.79 mm, with a low overall soil water deficit. The four vegetation types showed soil water deficits in different soil layers, with the lowest soil water deficits (0.34–0.52 mm) for SC, HR, and CF at 40–60 cm, and the lowest (0.65 mm) for CK at 160–180 cm. The soil water deficits of SC at 20–30 cm, CK at 0–10 cm, HR at 60–80 cm and CF at 180–200 cm were the highest (about 0.65 mm). SC, HR and CF had the lowest soil water deficit at 40–60 cm (0.34–0.52 mm) and CK had the lowest soil water deficit at 160–180 cm (0.65 mm). SC had the highest soil water deficit at 20–30 cm, CK at 0–10 cm, HR at 60–80 cm, and CF at 180–200 cm (about 0.90 mm).

#### Relationship between different soil water types

**Fig. 8:**
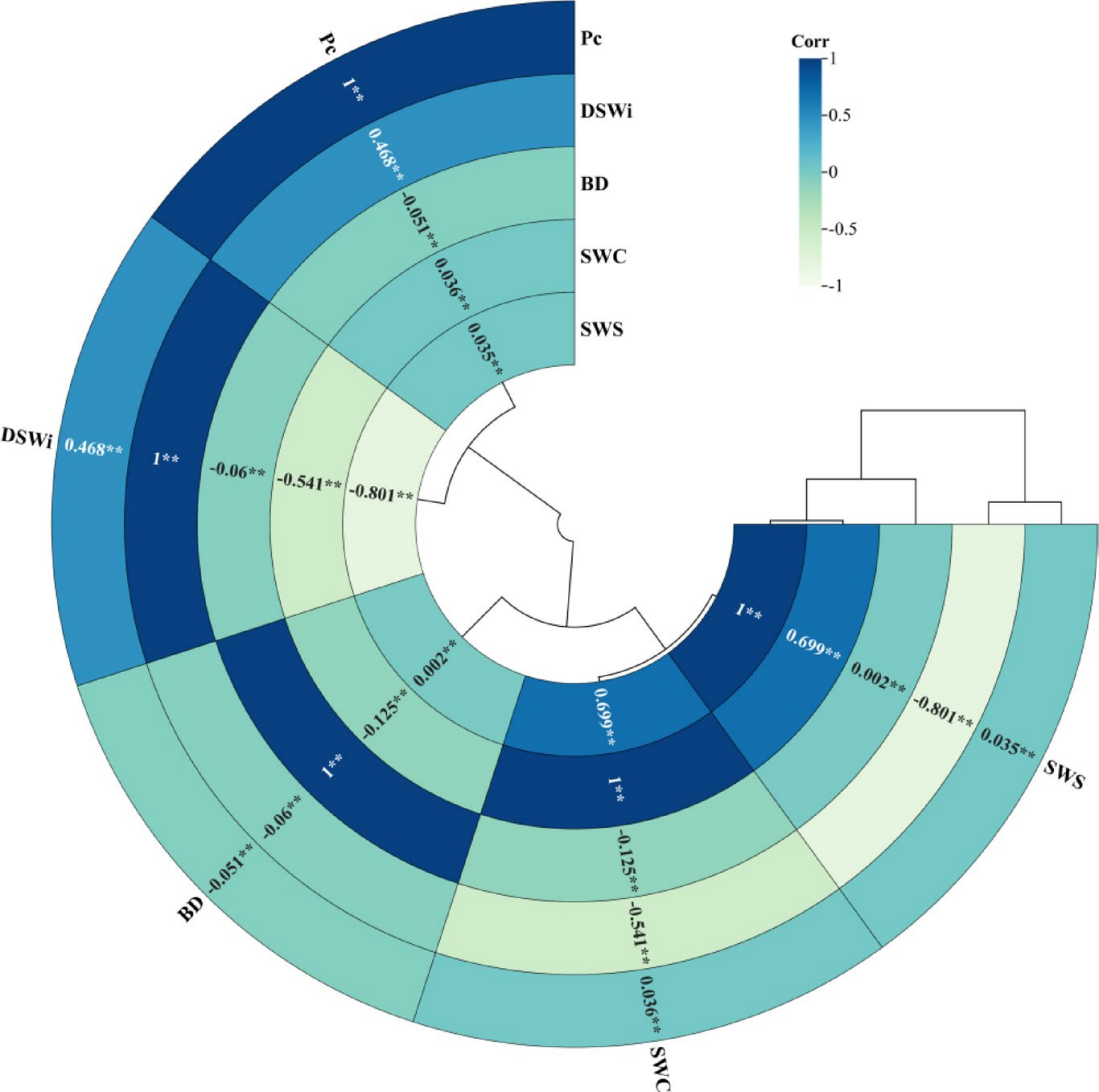
Hierarchical Pearson correlation analysis and clustering tree diagram for soil spatial moisture characteristics.

The relationships between soil water content (SWC), soil bulk density (BD), soil capillary porosity (Pc), soil water storage (SWS), and soil water deficit (DSWi) are shown in Fig. 8, which reveals that there were differences in the impacts on the four vegetation types, with the positive impacts being, in descending order, soil capillary porosity (Pc), soil water deficit (DSWi), soil bulk density (BD), soil water content (SWC), and soil water storage (SWS). Correlation analysis and construction of a clustered tree diagram showed that there was a significant correlation between them (*P* < 0.01). Soil water storage (SWS) had highly significant positive and negative correlations (*P* < 0.01) with correlation coefficients of (-0.801, 0.699) on soil water content (SWC) and soil water deficit (DSWi). In addition, according to the Pearson correlation analysis in Table 2, soil water content (SWC) and water storage (DSWi) were in highly significant negative correlation (*P* < 0.01) with a correlation coefficient of (-0.541). Some correlation also existed between the other parameters, but the correlation coefficients were relatively low.

**Table 2:** Hierarchical pearson correlation analysis of Spatial soil moisture characteristics.

指标 Norm	SWC	BD	Pc	SWS	DSWi
SWC	1				
BD	− 0.125	1			
Pc	0.036	− 0.051	1		
SWS	0.699**	0.002	0.035	1	
DSWi	− 0.541**	− 0.06	0.468**	− 0.801**	1

Pc: Soil capillary porosity; DSWi: Soil water deficit; BD: Soil bulk density; SWC: Soil water content; SWS: Soil water storage. ** stands for *P* < 0.01.

### Relationship between single fractal and multivariate dimensions of soil particle size distribution and soil spatial moisture

**Fig. 9:**
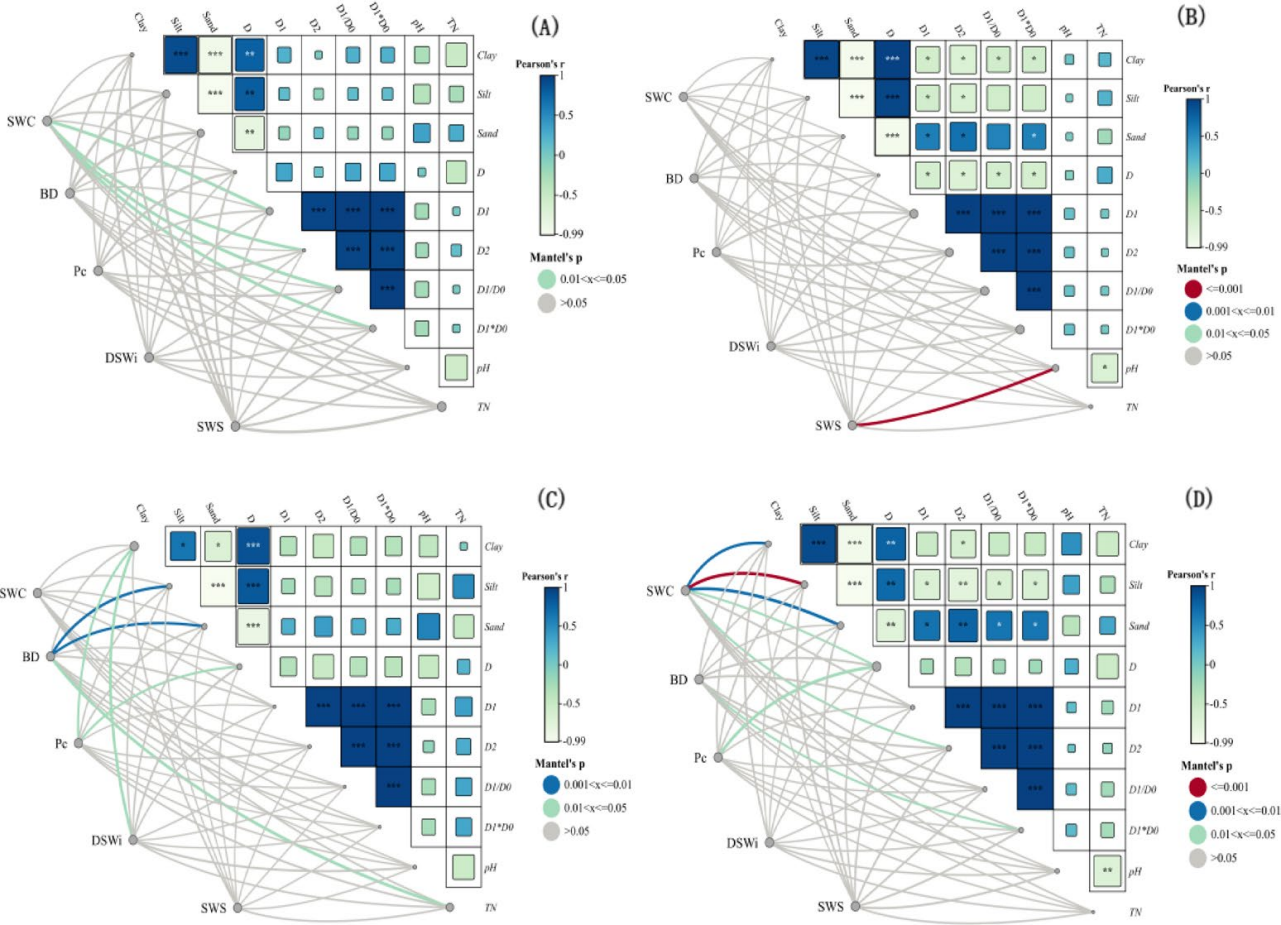
Pearson correlation and factor analysis of soil environmental variables and soil spatial moisture changes in different vegetation types. (**A**)–(**D**) represent four vegetation types, respectively. *** represents *P* < 0.001; ** represents *P* < 0.01; * represents *P* < 0.05.

The single and multiple fractal dimensions of the four vegetation types (SC, CK, HR and CF) of the soil were closely related to soil spatial moisture. Figure 9 A, the study of SC indicated that four parameters, soil D_1_, D_2_, D_1_/D_2_ and D_1_*D_2_, could significantly explain the variation of soil water content (SWC) (*P* < 0.05), while the relationship between other soil water parameters and soil particle size distribution was relatively weak. Figure 9B, CK indicated that there was a super significant correlation (*P* < 0.001) between soil water storage (SWS) and soil pH. Figure 9 C, HR indicated that there was a highly significant correlation (*P* < 0.01) between soil particle size silt and sand composition and soil bulk density (BD); Meanwhile, soil clay composition and single fractal dimension (D) were significantly correlated with the soil capillary porosity (Pc) and soil water deficit (DWS_i_) (*P* < 0.05); It is worth noting that the soil clay composition was also significantly (*P* < 0.05) correlated with the DWS_i_. Moreover, TN content was significantly correlated with soil bulk density (BD) (*P* < 0.05). Figure 9D, CF indicated that there was a super-significant correlation (*P* < 0.001) between soil water storage (SWS) and silt composition, and it was also highly significantly correlated (*P* < 0.01) with clay and sand composition. Also, soil water storage (SWS) was significantly correlated (*P* < 0.05) with soil single fractal dimension (D) and entropy dimension (D_1_); Soil bulk density (BD) was also significantly correlated (*P* < 0.05) with D_1_*D_2_, and soil single fractal dimension (D) was correlated (*P* < 0.05) with soil capillary porosity (Pc) significantly.

## Discussion

### Effect of different vegetation types on single fractal and multivariate dimensions

Vegetation can lead to changes in soil particle size fractal characteristics by affecting soil texture, and differences in vegetation types can indirectly affect soil particle size fractal parameters^26^. Soil particle sizes of the four vegetation types had the largest content of sand particles, followed by silt particles and the lowest content of clay particles. The highest content of sand willow clay particles at 180–200 cm was due to the well-developed root system of sandy willow, in which the fine particle sizes were leached to the deeper soil layer due to years of precipitation^27,28^, resulting in the elevated content of fine particle sizes in this soil layer. The content of CK and CF in the surface soil is relatively high because a large amount of litter has been accumulated for many years, which increases the content of soil organic matter and is conducive to the formation of fine particles^29^, resulting in a higher clay content in the surface soil than in the deep soil. Moreover, the fine particle sizes in CF increased with the depth of the soil layer, which further verified the top-down leaching process of fine particle sizes. Soil single fractal dimension (D) can characterize the overall roughness of soils of different vegetation types, and it has been pointed out that the D of fine-textured soils ranged from 2.60 to 2.80^30^, and that of coarse-textured soils ranged from 1.83 to 2.64, and that the D of SC, CK, HR, and CF faggots in the present study ranged from 2.01 to 2.14, 2.02–2.12, 1.97–2.09 and 1.96–2.09, and the D values of SC and CK were significantly greater than those of HR and CF, indicating that SC and CK have stronger soil water-holding capacity and nutrient retention capacity. Diao^31^ et al. studied the single fractal characteristics of soil particle sizes of different vegetation types in the source area of the Heihe River, and the results also showed that the soil particle sizes of different vegetation types had obvious fractal characteristics. The four vegetation types D-values were positively correlated with soil clay and silt content, and negatively correlated with sand content, a finding that is the same as that of Gao^32^ who studied the distribution of soil particle sizes in the active layer of the Tibetan Plateau, suggesting that the clay and silt particle sizes resulted in a more homogeneous distribution of soil particle sizes. The generalized dimension spectrum D(q) (Fig. 3) quantifies the complexity and non-uniformity of particle size distribution in different soil layers, and the D(q)-q curves show an inverse “S” decline and are more sensitive in the sparse zone, similar to the results of Chen^33^, which showed significant variations between the negative values of q. All four vegetation types have a D0 of 0.177, indicating that they all have the same soil particle size distribution. D_1_ and D_2_ reflect the concentration of soil particle size distribution and the variability of particle size. D_1_ and D_2_ were higher in CF than in SC, CK, and HR at 0–120 cm and 180–200 cm, and HR was higher than the other vegetation types at 120–200 cm, which indicated that the soil particle size fractal structure was finer in CF and HR.

### Effects of different vegetation types on characteristics of soil Spatial moisture

As important soil physical factors that can effectively reflect the physical conditions of soil water and structure, soil water content and soil bulk density can affect plant root penetration, soil nutrient retention, and movement in the soil, which in turn affect the structure and function of the ecosystem. In this study, the soil water content of HR was significantly higher than that of other vegetation at 0–40 cm soil depth, probably because most of the root system grows in the top layer of the soil, which is easy to block the soil pores and form a dense and very fine network, preventing the downward movement of soil water. Therefore, the soil water content of the surface layer is higher than other soil layers. Wang^34^ investigated the soil water content of CK and HR at 0–60 cm under different ecological restorations in the Loess Plateau of northern Shaanxi, and the soil water content was also significantly higher than that of other soil layers. The size of the soil bulk density further reflects the soil structure and aeration and water permeability. At the depth of 0–10 cm, the soil bulk density showed a trend of CK > CF > SC > HR, indicating that the soil in CK was compact and poorly aerated, whereas the soil in HR was loose and porous, with good aeration and permeability, which was conducive to the growth of plants. The size of soil porosity determines the content of soil water and air as well as the good or bad permeability^35^, and the overall porosity was not significant, indicating that the four vegetation types of soils had the same function of soil water containment. The four vegetation types soils consistently showed a trend of increasing, then decreasing, and increasing soil water storage, indicating that the vegetation absorbed more water in the surface and middle layers of the soil, while in the deeper layers of the soil, the absorption of water was not obvious, indicating that these layers are the vegetation types to absorb the soil moisture main soil layers^23^. SC, HR, and CF had a smaller water deficit at 40–60 cm and CK at 160–180 cm, which was due to the fact that the root interspersed of the four vegetation types improved the microenvironment such as soil pore structure, which increased the rate of water infiltration, and then effectively replenished the soil water in different soil layers^36^, which was similar to the findings of Liu^37^, that the distribution of the root system only to some extent determines their degree of water absorption.

### Influence of characteristics of soil spatial moisture on single and multivariate fractals of particle size distributions

Vegetation can inhibit soil erosion and enhance soil quality by optimizing the composition of soil particle sizes, for example, by increasing the ratio of clay to silt particle sizes. Soil particle sizes, on the other hand, provide the necessary resources for the growth and development of vegetation types by retaining water and nutrients^38^. In this study, factor analysis revealed that the variation of spatial distribution of soil moisture was not only determined by the composition of soil particle sizes, but also affected by the dispersion and uniformity of soil particle size distribution. Specifically, there was a strong link between the particle sizes D_1_, D_2_, D_1_/D_2_, and D_1_*D_2_ of the soil in which the SC was located and the soil water content, which was attributed to the promotion of water retention and transport by good soil structure. Soil water storage in lemon-growing sites is closely related to soil pH because changes in pH can affect the interaction forces between soil particle sizes, which in turn regulate the water retention properties of the soil. For HR and CF, the composition of silt, sand, and clay particles in the soil showed a significant correlation with soil bulk density, soil capillary porosity, and soil water deficit, mainly because silt and sand particles have a larger surface area with a stronger surface adsorption capacity^39^, and are therefore more likely to adsorb and retain water in the soil. This paper focuses on the regulation mechanism of water spatial variation on soil particle fractal under different vegetation types, but it is necessary to point out that soil mechanical properties, the influence of particle gradation fractal structure on soil deformation under impact load^40,41^ and biological cementation^42,43^ and other factors indirectly affect the water transport process by changing the stability of soil structure. For example, Yang^40^ et al. have shown that particle breakage can reshape the soil pore network, and biocementation technology can enhance the erosion resistance of surface soil^42^.These processes are potentially related to the correlation between fractal dimension and water content observed in this study. However, due to the limitations of research objectives and experimental design, the above mechanical and cementation effects have not been included in quantitative analysis. Future research can further integrate fractal theory, mechanical model, soil-structure interface constitutive relationship under cyclic loading and biological cementation control methods, reveal the interaction mechanism of vegetation-soil-hydrology-mechanics from the perspective of multi-physical field coupling^43^, and provide a more systematic theoretical framework for sandy land ecological restoration.

## Conclusion

This study analyzed the relationship between soil fractal parameters and water spatial dynamics under four typical vegetation types (*Salix cheilophila*, *Caragana korshinskii*, *Hippophae rhamnoides*, and *Corethrodendron fruticosum*) in the Mu Us Sandy Land, and the following conclusions were drawn: (1) The soil in the study area is dominated by sand, and the degree of change is higher than that of fine particles (clay and silt). The soil fractal dimension (D) was positively correlated with the content of clay and silt, and negatively correlated with the content of sand, and the particle gradation trend of the four vegetation in the 140–200 cm soil layer was consistent. The generalized dimensional spectral analysis shows that the fractal parameter D_0_ of the dense distribution area of soil particles is 0.177, indicating that the distribution range of soil particles of these vegetation is the same. (2) HR significantly increased the surface soil water content due to the well-developed shallow root system (0–40 cm), and the 120–200 cm soil layer was not reached by the root system, so the water storage in this soil layer was higher than in other soil layers; Whereas, SC and HR were consistent in the same soil layer, and the highest was at 160–180 cm. The bulk density and capillary porosity of CF and CK were not significant in the same soil layer, and the water content of CF was significant, showing a trend of increasing first and then decreasing. Among the four vegetation types, HR had the highest soil water storage (791.61 mm) and CK had the lowest (454.89 mm), which was directly related to the ability of the root system to improve porosity.The water deficit was the most serious (about 0.90 mm ) at 20–30 cm, 0–10 cm, 60–80 cm and 180–200 cm, respectively. (3) Soil particle fractal parameters D_1_, D_2_ and their combinations (D_1_/D_2_, D_1_*D_2_) can effectively characterize the spatial variability of soil moisture. Among them, there were significant correlations between D_1_, D_2_, D_1_/D_2_ and D_1_*D_2_ and soil water content in SC. There was a significant correlations between soil pH and soil water storage. There is a correlation between the composition of soil silt, sand and clay and soil bulk density, capillary porosity and water deficit. The soil particle composition of CF was significantly correlated with soil water storage, and the fractal parameters of soil particle distribution were significantly correlated with soil bulk density, capillary porosity, and water storage, indicating that the particle composition indirectly regulated the water retention ability through the fractal structure. This study reveals the coupling mechanism of vegetation type-fractal characteristics-water dynamics, which can provide a theoretical basis for vegetation configuration and soil hydrological function restoration in Mu Us Sandy Land.

## Data Availability

The datasets used and/or analysed during the current study available from the corresponding author on reasonable request.
